# Multifacility Outbreak of NDM/OXA-23–Producing *Acinetobacter baumannii* in California, 2020–2021

**DOI:** 10.1017/ash.2021.155

**Published:** 2021-07-29

**Authors:** Diana Holden, Tisha Mitsunaga, Denise Sanford, Tanya Fryer, June Nash, Emily Schneider, Rituparna Mukhopadhyay, Erin Epson, Matthew Sylvester

## Abstract

**Background:** NDM/OXA-23 carbapenemase-producing *Acinetobacter baumannii* isolates have been reported worldwide, but rarely in the United States. A California acute-care hospital (ACH) A identified 3 patients with pan-nonsusceptible *A. baumannii* during May–June 2020, prompting a public health investigation to prevent further transmission among the regional healthcare network. **Methods:** A clinical isolate was defined as NDM/OXA-23–producing *A. baumannii* from a patient at ACH A or B, or an epidemiologically linked patient identified through colonization screening during May 2020–January 2021. ACHs A and B are sentinel sites for carbapenem-resistant *A. baumannii* surveillance through the Antibiotic Resistance Laboratory Network (AR Lab Network), where isolates are tested for carbapenemase genes. The California Department of Public Health with 3 local health departments conducted an epidemiological investigation, contact tracing, colonization screening, and whole-genome sequencing (WGS). **Results:** In total, 11 cases were identified during May 2020–January 2021, including 3 cases at ACH A during May–June 2020, and 8 additional cases during November 2020–January 2021: 5 at ACH A, 1 at ACH B, and 2 at skilled nursing facility (SNF) A. Isolates from ACHs A and B were identified through testing at the AR Lab Network. Of the 11 patients (including the index patient), 4 had exposure at SNF A, where 2 cases were identified through colonization screening. Screening conducted at ACH A and 5 other long-term care facilities (LTCFs) identified no additional cases. WGS results for the first 8 cases identified showed 2–13 single-nucleotide polymorphism differences. Antibiotic resistance genes for all isolates sequenced included NDM-1 and OXA-23. On-site assessments related to a COVID-19 outbreak conducted at ACH A identified infection control gaps. **Conclusions:** Hospital participation in public health laboratory surveillance allows early detection of novel multidrug-resistant organisms (MDROs), which enabled outbreak identification and public health response. A high COVID-19 burden and related changes in infection control practices have been associated with MDRO transmission elsewhere in California. This factor might have contributed to spread at ACH A and hampered earlier screening efforts at SNF A, likely leading to undetected transmission. Extensive movement of positive patients among a regional healthcare network including at least 6 ACHs and 7 LTCFs likely contributed to the prolonged duration of this outbreak. This investigation highlights the importance of enhanced novel MDRO surveillance strategies coupled with strong infection prevention and control practices as important factors in identifying outbreaks and preventing further transmission in regional networks.

**Funding:** No

**Disclosures:** None

